# Novel Applications of Metabolomics in Personalized Medicine: A Mini-Review

**DOI:** 10.3390/molecules22071173

**Published:** 2017-07-13

**Authors:** Bingbing Li, Xuyun He, Wei Jia, Houkai Li

**Affiliations:** 1 Center for Traditional Chinese Medicine and Systems Biology, Institute for Interdisciplinary Medicine Sciences, Shanghai University of Traditional Chinese Medicine, Shanghai 201203, China; libingbingxp@163.com (B.L.); hexuyun1993@126.com (Y.H.); 2 Cancer Epidemiology Program, University of Hawaii Cancer Center, Honolulu, HI 96813, USA; 3 Center for Translational Medicine, and Shanghai Key Laboratory of Diabetes Mellitus, Shanghai Jiao Tong University Affiliated Sixth People’s Hospital, Shanghai 200233, China

**Keywords:** metabolomics, personalized medicine, interindividual variability, pharmaco-metabonomics, patient stratification

## Abstract

Interindividual variability in drug responses and disease susceptibility is common in the clinic. Currently, personalized medicine is highly valued, the idea being to prescribe the right medicine to the right patient. Metabolomics has been increasingly applied in evaluating the therapeutic outcomes of clinical drugs by correlating the baseline metabolic profiles of patients with their responses, i.e., pharmacometabonomics, as well as prediction of disease susceptibility among population in advance, i.e., patient stratification. The accelerated advance in metabolomics technology pinpoints the huge potential of its application in personalized medicine. In current review, we discussed the novel applications of metabolomics with typical examples in evaluating drug therapy and patient stratification, and underlined the potential of metabolomics in personalized medicine in the future.

## 1. Introduction

Interindividual variations either in therapeutic outcome or disease susceptibility are common in the clinic due to the complicated interactions between genetic and environmental factors [1,2]. Accordingly, the concept of personalized medicine (or precision medicine [3]) arouses great interest currently in the context of revolutionized innovations in biomedical research and high throughput analytical technology. The ultimate goal of personalized medicine is to enable clinicians to prescribe the right medicine to the right patient at the right time with maximum efficacy and minimal toxicity, or to predict the susceptibility to disease onset among populations in advance. To achieve these goals, great efforts have been paid to correlate drug responses with host genetic polymorphisms, i.e., pharmacogenomics [4]. Although there have been some impressive achievements in pharmacogenomics during the past decades [5], pharmacogenomics is blind to the impact of environmental elements, as well as the co-metabolism of host and gut microbiota [3], which plays important roles in determining drug metabolism and disease formation.

At the end of the 20th century, metabolomics/metabonomics which is to analyze the variation of endogenous metabolites in response to modifications by biological system such as cells, tissues and body fluids (see review [3] for clear definitions), came into sight following genomics, transcriptomics and proteomics. Metabolomics is the integrative readout of both genetic and environmental impacts, and has been successfully applied in a variety of studies such as biomarker discoveries [6,7], mechanistic study of disease and drug activity [8,9,10], drug-induced toxicity [11,12] and metabolism [13,14]. Moreover, the concept of pharmacometabonomics (a term interchangeable with pharmaco-metabolomics [3]) was proposed and defined as “the prediction of the outcome of a drug or xenobiotic intervention in an individual based on a mathematical model of pre-intervention metabolite signatures” by Nicholson et al. [15]. Pharmacometabonomics has been used to seek metabolic biomarkers that could potentially predict different responses of clinical drugs by identifying differential metabolites at baseline and correlating their variations with the therapeutic outcomes. Similarly, metabolomics has also been applied for evaluating the individual susceptibility to diseases among populations by integrating their baseline metabotypes (metabolic profiles) in a cohort with the risk of disease occurrence contributing to realization of patient stratification.

In the current mini-review, we first gave a brief description on frequently used analytical instruments for metabolomics such as nuclear magnetic resonance (NMR), gas chromatography−mass spectrometry (GC-MS) and liquid chromatography−mass spectrometry (LC-MS). Then, we primarily focused on discussing the novel applications of metabolomics in predicting drug responses and patient stratification, which sheds light on the prospect of metabolomics in personalized medicine in the near future.

## 2. Metabolomic Platforms

Metabolomics is a relatively young discipline compared to other well-established “-omics” such as genomics, transcriptomics and proteomics. The rapid development of metabolomics is due to the dramatic advances in analytical instrumentation and coupled data mining techniques during the past ten years. There are three main metabolomic platforms, including NMR, GC-MS and LC-MS. Given the different analytical coverage of these three platforms, they have been used for targeted/untargeted metabolomic studies either individually or in combination for detecting metabolites such as amino acids, organic acids, lipids and sugars. It is well-recognized that each platform has its own advantages and disadvantages in metabolomic study. Generally, NMR is superior in metabolite identification, quantitation and automation, but with lower sensitivity and higher start-up cost compared to MS-based metabolomic approaches. GC-MS is relatively robust, with good sensitivity, convenient metabolite identification by using commercial databases and software, but sample preparation is labor-intensive and novel compound identification is difficult. LC-MS has much higher sensitivity, wider metabolite detection coverage and diversified methodology, but less robustness and more difficult compound identification compared to NMR or GC-MS. The comprehensive comparisons on advantages and disadvantages of these platforms have been made by some reviews [16,17,18].

## 3. Metabolomics in Predicting Drug Responses

### 3.1. Aspirin

Aspirin, with its potent analgesic, antipyretic, anti-inflammatory and anti-platelet effects, is one of the most widely used drugs. However, there are about 25% of high-risk patients who are aspirin-resistant in terms of platelet reactivity and atherothrombotic events. Yerges-Armstrong et al. [19] investigated the metabolic mechanisms underlying aspirin resistance by correlating the pre-dose metabolic signatures with interindividual variations after aspirin therapy in the Heredity and Phenotype Intervention (HAPI) heart study. They selected 76 healthy volunteers (40 good-responders and 36 poor-responders with the criteria of a change in collagen-induced ex vivo platelet aggregation) who underwent two weeks of aspirin treatment. Their pre- and post-dose serum samples were profiled with GC/MS-based untargeted metabolomics. First, 18 differential metabolites were identified in post-dose samples from all 76 subjects, and the purine metabolism pathway was found to be significantly affected by aspirin. The metabolites in the purine metabolism pathway were compared between good and poor responders at both pre- and post-dose of aspirin. It showed that inosine levels were elevated by aspirin in both good- and poor-responders, but the levels in post-dose patients were higher in poor-responders than that in good-responders. Then, the identified drug response-related metabolites were further validated in another group of subjects (19 good-responders and 18 poor-responders) from the same study.

Following the metabolomic analysis, authors further explored the genetic association of purine metabolism-related genes by using a “pharmacometabolomics-informed pharmacogenomics” approach. The association analyses were performed between single-nucleotide polymorphisms (SNPs) in the 9-purine metabolism-related genes and ex vivo platelet aggregation. They found 51 SNPs in one of the purine metabolism genes, adenosine kinase (ADK), that was strongly associated with changes in platelet aggregation during aspirin intervention, and the strongest SNP was the intronic variant rs16931294. Less G allele was associated with higher platelet aggregation after aspirin exposure than the common A allele. Moreover, the G allele of rs16931294 was significantly associated with higher levels of AMP, xanthine and hypoxanthine at pre-aspirin, as well as inosine and guanosine at post-dose of aspirin therapy compared to those in the A allele [19]. It was concluded that alteration of metabolites in purine metabolism pathway was involved in interindividual variations of aspirin efficacy, while the integration of pharmacometabolomics with pharmacogenomics could deepen the understanding on the mechanism of interindividual variation in drug responses at both genetic and metabolic levels.

In addition to the untargeted metabolomics, a targeted metabolomic study was also performed by analyzing primary and secondary amines [20]. A total of 19 differential metabolites were identified between post- and pre-dose aspirin intervention in a discovery cohort of 80 subjects (42 subjects from the first quartile and 38 from the fourth quartile based on collagen-induced aggregation). Then, these differential metabolites were compared between the two extreme quartiles of aspirin responses, and the variations of four metabolites were correlated with drug response, i.e., alanine, taurine, glycine and serotonin. These four metabolites were further validated in an independent replication cohort of 125 subjects, in which only serotonin levels were consistently higher in the fourth quartile (19 subjects) than in the first quartile (19 subjects) at both pre- and post-dose of therapy. The predicting power of serotonin was confirmed with an ex vivo agonist-induced platelet aggregation test and an independent cohort of 38 healthy subjects with lowest or highest baseline serotonin levels in platelet-rich plasma. The results showed that collagen-induced platelet aggregation was inhibited to a lesser extent in subjects with higher baseline serotonin levels. Accordingly, the baseline level of serotonin was a potential predictor for determining the different responses towards aspirin therapy in patients.

### 3.2. Simvastatin

Statins are classical HMG-CoA reductase inhibitors that are widely used for reducing plasma LDL-cholesterol (LDL-c) and thus the risk of cardiovascular disease. However, more diversified biological activities of statins have been reported such as anti-inflammatory effects and modulation of immunity [21], leading to either therapeutic benefits or serious side effects including type 2 diabetes mellitus and myopathy [22]. Moreover, there are obvious interindividual variations in the therapeutic efficacy of simvastatin.

Kaddurah-Daouk et al. compared the baseline and post-dose lipid profiles with targeted lipidomics between good and poor responders (24 subjects in each group) with respect to the percentage change in LDL-c reduction or the secondary outcome C-reactive protein (CRP) in the Cholesterol and Pharmacogenetics (CAP) study [23]. There were about 40 metabolites that were significantly changed in good responders, but not in poor responders. Thirteen saturated or monounsaturated fatty acids were increased, and 15 polyunsaturated fatty acids (PUFAs) were decreased in good responders. Further analysis revealed that the baseline concentrations of n-6 and n-3 were positively correlated with LDL-c reduction, while CE and DG SFA were negatively correlated. DG-n6 and FA-n3 were positively correlated with the treatment outcome. The baseline levels of PE plasmalogens positively, while PC plasmalogens negatively correlated with CRP changes after therapy. These results indicated that the baseline lipid signatures were potential biomarker for predicting different outcomes of simvastatin treatment.

Meanwhile, they observed lower baseline plasma levels of several primary and secondary bile acids were strongly correlated with the effect on LDL-c reduction during simvastatin treatment in the randomly selected subjects, including higher baseline levels of LCA, TLCA, GLCA and coprostanol (COPR) in good responders [24]. Since the secondary bile acids are derived from gut microbiota, the results shed light on the potential role of gut microbiota in affecting simvastatin efficacy. Our recent study confirmed that the therapeutic efficacy of simvastatin was attenuated by gut microbiota modulation with antibiotic through suppressing the bile acids synthesis from cholesterol [25].

Within the same CAP study, an untargeted metabolomic profiling was also performed to investigate the correlation between baseline metabotypes and LDL-c reduction after simvastatin treatment [26]. The results showed that lower levels of uridine, xanthine, 2-hydroxyvaleric acid, succinic acid, stearic acid, and higher levels of pseudouridine, galactaric acid at baseline were correlated with the reduction of LDL-c upon simvastatin treatment. These identified pre-dose variations of metabolites between good and poor responders could not only be potential predictors for evaluating the treatment outcome of simvastatin, but reveal novel mechanisms underlying different responses to simvastatin therapy.

### 3.3. Acamprosate

Acamprosate is an amino acid derivative that is approved for the treatment of Alcohol Use Disorders (AUDs) [27]. However, only a sub-group of AUDs patients are sensitive to acamprosate therapy [28]. As a result, it is critical to find biomarkers to predict the therapeutic outcomes of acamprosate treatment among AUDs patients in the clinic.

Nam et al. [29] analyzed the baseline and post-treatment metabotypes in a group of 120 alcohol-dependent subjects including 71 responders and 49 non-responders during 12 weeks of acamprosate treatment, by using a pharmacometabolomic approach. The authors first profiled 36 metabolites in the serum samples both at baseline and post-treatment of acamprosate in a discovery cohort (51 responders and 39 non-responders) by using UPLC-MS/MS. First of all, 14 differential metabolites were identified between the baseline and post-treatment of acamprosate. In the replication cohort, 4 out of 14 differential metabolites showed similar changes with that in the discovery cohort, in which glutamate was notably changed. The levels of glutamate were higher in the baseline of responders than in non-responders, and reduced greatly in responders, but not in non-responders after acamprosate treatment. Interestingly, the baseline levels of ammonia were also higher and reduced after acamprosate treatment in responders. Moreover, multivariable logistic regression indicated that a higher baseline glutamate or ammonia were good predictors of responses towards acamprosate treatment. In the following animal study, the authors revealed that acamprosate accelerated glutamine synthesis from glutamate and ammonia by stimulating glutamine synthetase, which accounted for the reduction of glutamate after acamprosate treatment. Accordingly, the baseline levels of glutamate could be a potential biomarker for predicting the therapeutic outcomes of acamprosate in the clinic.

### 3.4. Antihypertensive Drugs

Hypertension is one of the most common chronic diseases, which affects about one billion people worldwide [30]. Although many types of antihypertensive drugs are available, only about 40% of hypertensive patients have optimally controlled blood pressure [31]. It is well recognized that the efficacy of antihypertensive drugs varies greatly between individuals and even races. However, little is known about the mechanisms underlying the different responses of individuals to antihypertensive drugs. Currently, the emerging pharmacometabolomics discipline is helping to reveal the reasons for the variations in drug efficacy.

Atenolol belongs to the beta-receptor blocker family and is a classical antihypertensive drug with obvious racial and individual differences. Wikoff et al. [32] investigated the biochemical changes induced by atenolol in Caucasians and African-Americans by using a GC-TOF metabolomics platform. A total of 272 subjects with high blood pressure were included, in which their plasma samples at baseline and 9 weeks after atenolol treatment were collected for metabolomic analysis. Significant differences were found between Caucasians and African Americans in the degree of blood pressure reduction after atenolol treatment, as well as metabolic differences including decreased saturated, monounsaturated and polyunsaturated free fatty acids in Caucasians. Moreover, their results also revealed a genetic variation of the gene that encodes lipases contributing to the racial differences in the response to atenolol therapy.

In addition, a metabolomic assessment of the individual variations to antihypertensive drugs, including atenolol and hydrochlorothiazide, was performed with non-targeted metabolomics approach in white and black subjects [33]. Plasma samples at baseline and post-treatment from 128 white and 109 black subjects for atenolol, and 123 white and 83 black subjects for hydrochlorothiazide therapy were collected. As expected, although the majority of the hypertensive subjects experienced an obvious reduction in blood pressure, there were significant differences in blood pressure reduction between white and black subjects with both treatments. The metabolomic analysis revealed that the baseline levels of 5-methoxytryptamine were negatively correlated with the change in blood pressure of white participants in atenolol-treated subjects, while seven metabolites at baseline were associated with that in black participants. The baseline levels of arachidonic acid and another unknown metabolite (223548) were positively correlated with the change in blood pressure of white participants with hydrochlorothiazide treatment, whereas the metabolite (223548) was negatively associated in black participants. On the basis of baseline metabolomic profiles, multivariable models were constructed to predict the antihypertensive response for all participants treated with either atenolol or hydrochlorothiazide. Statistically significant results were obtained in the multivariable models from both the discovery and validation datasets, and increased predictive power was observed in models with increased numbers of subjects. The subsequent analysis revealed a number of metabolic pathways that were altered jointly or individually in both white and black participants after antihypertensive drug therapy implying mechanisms underlying the different outcomes of drug therapy. A brief summary of Metabolomics-based studies on drug efficacy was provided in Table 1.

## 4. Metabolomics in Identifying Biomarkers of Disease Susceptibility

In addition to the different responses upon identical drug therapy among subjects with same disease, individual susceptibility to diseases also varies significantly among population because of diversified genetic or metabolic backgrounds. Therefore, it is of great significance for personalized medicine to identify biomarkers for predicting disease susceptibility among subjects.

### 4.1. Variations in Branched-Amino Acids Predicted Risk of Diabetes

Metabolic diseases are chronic consequences of dysregulation of energy homeostasis and obesity is the most common risk factor. The metabolic alterations are usually present for years prior to the development of metabolic disease, while individual susceptibility to metabolic disease varied greatly even among obese subjects. Interestingly, about one-third of obese subjects are free of any metabolic disorders according to a recent meta-analysis [34]. It is therefore of great significance to identify metabolic biomarkers that can sub-classify patients with different risks for disease development.

Wang et al. [35] performed a nested case-control metabolomic analysis in the Framingam Offspring Study, in which baseline metabolic profiles were analyzed in both 189 new-onset diabetes subjects during a 12-year follow-up period and propensity-matched controls, respectively. Using paired analysis, they identified five metabolites at baseline that were significantly different between cases and controls, including three branch-chained amino acids (leucine, isoleucine and valine), and two aromatic amino acids (phenylalanine and tyrosine). Further analysis indicated that the subjects with the top quartile plasma amino acids levels had at least a 2-fold higher chance of developing diabetes during the following 12 years, compared to those with the lowest levels of plasma amino acids, and this correlation was dramatically increased if only three branch-chained amino acids were included in the analysis. The predictive power of the identified amino acids for diabetes onset was confirmed in independent replication samples, as well as in a random population within the Framingham Offspring cohort. This study not only suggested the importance of amino acids in development of diabetes, but also highlighted the great potential for uncovering metabolic predictors for metabolic diseases by metabolomic analysis.

### 4.2. Variations in Free Fatty Acids Predicted Metabolic Consequences and Therapeutic Outcomes of Obesity

Free fatty acids (FFAs) are important substrates for energy metabolism that play crucial roles in the development of obesity and obesity-based metabolic diseases. In one of our recent publications [38], we performed a UPLC/Q-TOFMS-based targeted metabolomic analysis on 40 FFAs in the blood of four different groups of subjects with different metabolic statuses, i.e., normal weight (NW), overweight/obese metabolically healthy (HO) and overweight/obese diabetic (UO), to address the questions: (1) whether the FFA profiles are different among the three groups; (2) whether there are specific FFAs that are predictive for the development of UO from HO over a ten-year observation period; (3) whether specific FFAs are related to changes in metabolic markers in response to therapeutic intervention. First, a cross-sectional study consisting of 132 NW, 107 HO and 73 UO subjects was performed, in which a group of unsaturated fatty acids (USFAs) was positively correlated with metabolic markers. Among these USFAs, DGLA (C20:3, n6) exhibited the highest correlation with metabolic markers.

**Figure 1:**
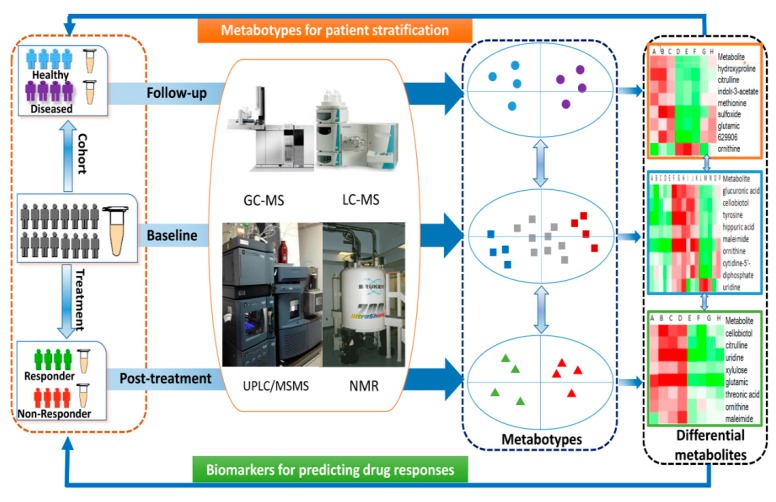
Schematic view of metabolomics in personalized medicine.

Additionally, we performed a longitudinal metabolomic analysis in another cohort consisting of 62 overweight subjects to evaluate whether the identified differential FFAs were predictive for the later development of metabolic syndrome. The selected 62 overweight subjects had normal metabolic markers at baseline, but 50 of them developed into UO and 12 remained HO after 10 years. Interestingly, the baseline metabolic markers between UO and HO were similar, but 6 FFAs at baseline were significantly higher or lower in the subset of UO subjects. These results confirmed that the baseline levels of certain FFAs were predictive for the future development of metabolic syndrome. To investigate further the correlation of FFAs with the outcome of therapeutic interventions, longitudinal analysis of FFAs in blood was conducted in 40 type 2 diabetic patients who received gastric bypass surgery and 38 obese subjects on an 8-week low carbohydrate diet. In both studies, similar strong correlations were found between UFAs and metabolic markers, and some of UFAs were proposed to be potential predictors for therapeutic responses to either metabolic surgery or dietary intervention. A schematic view of the application of metabolomics in personalized medicine was shown in Figure 1.

## 5. Conclusions and Perspectives

Metabolomics is a relatively new approach compared to other “-omics”. With the rapid development of analytical technology and increasing passion of precision medicine, metabolomics has been extensively trialed in both the clinical and experimental studies. The individual metabotype at the baseline is informative for predicting responses to drug therapy, and personal susceptibility to diseases during the follow-up. Meanwhile, the different metabolic profiles between baseline and post-treatment could reveal novel mechanisms of drugs by integrating the altered metabolites with involved metabolic pathways. In respect to the genetic and metabolic impacts on therapeutic outcomes, it is necessary to combine metabolomics with genomics for elucidating the mechanisms underlying different drug responses, such as the proposed GWAS-metabolomics strategy [39].

Currently, pharmacometabolomics is still in its infancy because most pharmacometabolomic studies are merely focused on revealing the correlation between baseline metabotypes with drug responses or disease susceptibility. Moreover, since the baseline metabotypes are usually influenced by factors such as diets, ages, drug intake and gut microbiota, these factors should be taken into account in designing pharmacometabolomic study to minimize the metabolic biases. For example, diet is one of the most influential factors for metabolome. It is necessary to keep the diets identical during the observation period or at least before the sample collection. In addition, since the variation of gut microbiota could also affect the metabotype of host, the integration of metabolomic and gut microbiome analysis will deepen the understanding on the roles of gut microbiota on host metabolism and responses to drug therapy. Although some identified differential metabolites are “exciting” because of their strong statistical correlation between baseline metabotypes and drug responses, further strict validations are critical for evaluating their reliability and reproducibility with large and independent clinical samples. Given the well-recognized advantages and disadvantages of each metabolomics platforms, it is necessary to either enlarge the “metabolic window” by combining different types of analytical instruments, or specifically quantify a group of targeted metabolites by adoption of commercial kits produced by companies.

On the other hand, little is known about the roles of these “biomarkers” in mediating drug responses or disease susceptibility currently, nevertheless, it is of great significance to understand the novel functions of individual metabolite or their combination under certain conditions by using interdisciplinary approaches. Although big challenges there are, the efforts of validating the predicting power of potential “biomarkers” and elucidating their roles will be a rewarding task to accelerate the realization of personalized medicine in clinic. We envisage that with the great passion on personalized medicine, metabolomics and pharmacometabolomics will be increasingly applied and developed in the coming years on the basis of technical innovation, as well as the integration with other omics approaches.

## Figures and Tables

**Table 1:** Brief summary of metabonomics-based studies on drug efficacy.

Drugs	Drug Efficacy	Metabolomics	Key Metabolite	Responses	Ref.
Aspirin	antiplatelet aggregation	GC-MS-based untargeted metabolomics	inosine	Increased in plasma of poor-responders at post-treatment	[19]
		MS-based targeted metabolomics	serotonin	Increased in plasma of poor-responders at both baseline and post-treatment	[20]
Simvastatin	reducing plasma LDL-cholesterol	GC/MS-based untargeted metabolomics	LCA, TLCA, GLCA, COPR	Increased in plasma of good-responders at baseline	[24]
		GC/TOFMS-based untargeted metabolomics	fructose	Increased in plasma of poor-responders at baseline	[26]
			xanthine, 2-hydroxyvaleric acid, succinic acid, stearic acid	Increased in plasma of good-responders at baseline	
Acamprosate	treating Alcohol Use Disorders (AUDs)	UPLC-MS/MS-based untargeted metabolomics	glutamate	Increased in plasma of good-responders at baseline	[29]
Atenolol	lower blood pressure	GC-TOFMS-based untargeted metabolomics	5-methoxytryptamine	Increased in plasma of good-responders at baseline	[33]
Hydrochlorothiazide	lower blood pressure	GC-TOFMS-based untargeted metabolomics	arachidonic acid, unknown metabolite (223548)	Increased in plasma of poor-responders at baseline	
			unknown metabolite (223548)	Increased in plasma of good-responders at baseline	
Sertraline	antidepressant	GC-TOFMS-based targeted metabolomics	5-Methoxytryptamine	Increased in plasma of good-responders at baseline	[36]
L-carnitine	treating sepsis	NMR-based untargeted metabolomics	acetylcarnitine/carnitine	Increased in plasma of poor-responders at baseline	[37]
